# Moderating role of job satisfaction on turnover intention and burnout among workers in primary care institutions: a cross-sectional study

**DOI:** 10.1186/s12889-019-7894-7

**Published:** 2019-11-14

**Authors:** Xuyu Chen, Li Ran, Yuting Zhang, Jinru Yang, Hui Yao, Sirong Zhu, Xiaodong Tan

**Affiliations:** 1 School of Health Sciences, Wuhan University, Donghu Road 115, Wuhcang District, Wuhan, 430000 Hubei China; 2 School of Clinical Medicine, Wuhan University of Science and Technology, 947 Heping Avenue, Qingshan District, Wuhan, 430000 Hubei China

## Abstract

**Background:**

Global countries are suffering from a shortage of health professionals. Turnover intention is closely related to job satisfaction and burnout, making good use of these relationships could alleviate the crisis. Our research aims to examine the mediating role of job satisfaction in the relationship between burnout and turnover intention.

**Methods:**

This research was conducted in Huangpi, China. The convenience sampling method and self-administereded questionnaires were used. 1370 of valid samples were collected with 97.72% effective rate. Descriptive analyses were conducted to describe social demographic factors. The structural equation model (SEM) was performed to adjust model fitting, and the mediation effect test was carried out by using the bootstrap method. Sobel-Z test was used to verify the significance of mediation effect.

**Results:**

The mean age was 36.98 (SD = 9.84). The fitting indices of hypothetical model are not good. After the adjustments, *χ*^2^/df = 5.590, GFI = 0.932, AGFI = 0.901, CFI = 0.977, NFI = 0.973, IFI = 0.977, TLI = 0.970, RESEA = 0.058. The revised model fitted well, and the SEM was put up by using the bootstrap method. The mediating effect is partial, and Soble-Z test indicates that the mediation effect is significant. Burnout is negatively correlated with job satisfaction (*p* < 0.01) and the standardized path coefficient is − 0.41. Job satisfaction is also negatively correlated with turnover intention (p < 0.01) and the standardized path coefficient is − 0.18. Burnout is positively correlated with turnover intention (p < 0.01) and the standardized path coefficient is 0.83.

**Conclusions:**

Job satisfaction is a mediating variable that affects the relationship between burnout and turnover intention. The mediating effect was a partial mediating effect and has a low impact of 7.4%. Improving treatment and giving more promotion opportunities for workers to improve job satisfaction, conducting career planning course and paying attention to employee psychological health to reduce job burnout. The above measures may be helpful to reduce employee turnover rate and alleviating the current situation of a shortage of health personnel in China.

## Background

Turnover is generally viewed as the movement of staff out of an organization. It was regarded as a two-dimensional concept, distinguishing between the act of leaving as voluntary or involuntary, and between the leaving and joining of an individual to an organization [1]. A previous study [2] defined turnover intention as the next withdrawal behavior when employees encounter dissatisfaction. Mobley et al. [3] pointed out that turnover intention was the intention of a worker to leave an organization deliberately after a period of time working in a particular organization, after careful consideration, which belonged to voluntary turnover. It is considered as an outcome of affective variables (such as burnout and job satisfaction) rather than actual turnover [4]. That is, turnover intention can predict actual turnover behavior.

An American clinical psychologist first proposed the term “job burnout [5]”. At present, job burnout was defined as the symptoms of practitioners in the service industry who were unable to cope effectively with the continuing pressure at work, included emotional exhaustion, depersonalization and reduced personal accomplishment [6]. Initially, job satisfaction was described as the physical and psychological satisfaction of staff in their work [7]. In later studies, it was defined as the realization of a person’s work values in the work situation, resulting in a pleasurable emotional state [8, 9]. The definition of “the attitude towards one’s work and related emotions, beliefs and behaviors” was used in our study, which not only depended on the nature of the work, but also the personality, attitude and expectation of medical staff [10, 11]. Low job satisfaction is the most common cause of staffs’ turnover and low quality of health care service.

There is a plethora of researches on job satisfaction, burnout and turnover intention. A study found that higher work pressure and lower job satisfaction could easily lead to burnout, that is, job satisfaction was one of the important predictors of burnout [12]. However, another study of American surgeons had the opposite result: burnout is the biggest predictor of job satisfaction [13]. In fact, satisfaction and burnout are in no particular order and they need to be determined on a case-by-case basis. Yin et al. [14] found that satisfaction with work itself, occupational risks and off-duty arrangements were the main factors affecting doctors’ job burnout through a survey on doctors in public hospitals. A study showed that job satisfaction had a direct predictive effect on burnout of middle school teacher, and it also existed as a mediating variable between job stress and job burnout [15]. Arie R and Yoram N had nosed out that negative correlation was existed in job satisfaction and job stress, burnout [16].

Most of researches supported that burnout had a positively strong predictive effect on turnover intention [17–22]. For example, emotional exhaustion, negative stagnation and other factors related to burnout have a significant positive correlation with turnover intention [23]. Beyond that, a study revealed that job satisfaction was negative correlated with turnover intention [24]. In the study of Guo et al. [25], turnover intention decreased with the increase of job satisfaction, this opinion was verified from a study of medical workers in Macao. And Wu et al. [26] also confirmed this result from a research of clinical nurses in Changsha. Therefore, the impact of job satisfaction on turnover intention is important, direct and negative. Satisfaction has a significant predictive effect on turnover intention.

At present, the medical working environment in China is tense, more and more medical personnel have left their jobs and changed their posts. As a result, there is a shortage of medical staff, which destroys the medical environment and disrupts the medical order and affects the health of the people. A study based on the National Health Service Survey in 2013 on China [27] found that the percentage of employees with low, medium and high turnover intentions was 44.5, 43.7 and 11.8% respectively in primary care institutions. And a foreign study [28] has investigated the turnover intention of nurses, and also found that 54% of them have turnover intention and 35% have turnover behavior. In view of the current shortage of medical personnel in various countries [29], job satisfaction and turnover intention may therefore be a crucial and topic concern.

Most previous research focused on the relationship between job satisfaction, turnover intention and burnout, and exploring the factors affecting the three. However, few studies focus on the mediating effect of a certain factor. Such research in China is still in its infancy. The purpose of the research is to examine the mediating role of job satisfaction in the relationship between burnout and turnover intention.

## Methods

### Participants and setting

A cross-sectional survey design was used. This quantitative research was conducted in Huangpi District, Wuhan, China. The investigation involved a total of 20 public primary care institutions in Huangpi, including 18 township health centers and 2 community health centers. It conducted from March 2019 to June 2019. The Convenience sampling method was used. Taking into account the scale and level of institutions in rural area, 25 copies of each institutions were determined. Sample size was amplified according to the inefficiency of 10%, and the final expected sample size was 550. Specialized investigators were trained to reduce information bias, inclusion and exclusion criteria was made to reduce selection bias. The inclusion criteria of participants were as follows, (1) Eligible participants had at least 6 months’ work experiences in their own workplace. (2) An employee who had not suffered from mental illness and had not been stimulated by major adverse life events in the near future. (3) Participants were voluntary. And we excluded staffs with a working time of less than 6 months, and employees who were not in the post during the investigation. A flow diagram of participants was shown in Fig. 1 in supplementary materials. There were 1402 questionnaires distributed. Questionnaires with uncompleted answers or suspected unreal answers were excluded. Finally, a total of 1370 of valid samples was collected with 97.72% effective rate. All responses were anonymous to protect the privacy of participants.

**Fig. 1:**
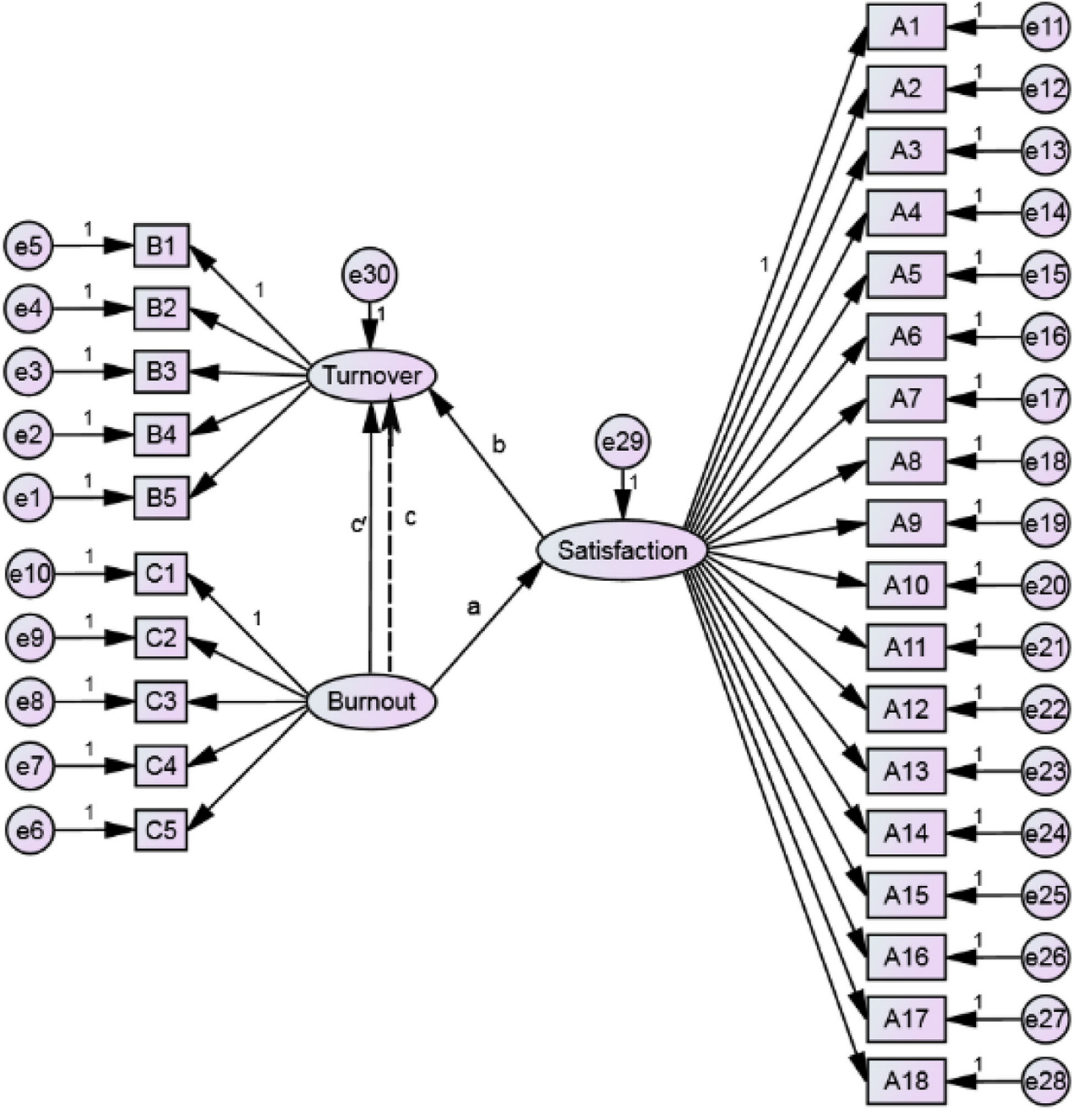
The hypothetical model of the relationship between job satisfaction, burnout and turnover intention

### Measures

We used self-administered questionnaires, which were classified into three parts, job satisfaction, burnout and turnover intention. Then we adjusted the questionnaire according to the pilot survey, actual situation and local culture. All the measures were followed the translation and back-translation process from English to Chinese [30]. The three scales all use the 5-point Likert scale. Content of each scale was shown in Table 1 in supplementary materials. The KMO measure and Bartlett’s spherical test were used to test construct validity, it was acceptable if values of KMO measure were greater than 0.50 and *p* value of Bartlett’s spherical test less than 0.05. Cronbach’s alpha coefficient was calculated to examine internal consistency reliability, values higher than 0.70 were considered satisfactory.

**Table 1:** Demographic characteristics of the respondents (*n* = 1370)

Variables	Group	n	%	Means *(SD* ^*a*^*)*
Gender	Male	426	31.09	–
	Female	944	68.91	
Age(year) ^**b**^	< 30	438	31.97	36.98 (9.84)
	30–39	305	22.26	
	40–49	472	34.45	
	> 49	155	11.31	
Occupation	Physician	586	42.77	–
	Nurse	562	41.02	
	Medical technician	92	6.72	
	Public health personnel	66	4.82	
	Pharmacist	34	2.48	
	Others	30	2.19	
Educational background	University and above	423	30.88	–
	College	636	46.42	
	High school/Technical school	286	20.88	
	Junior high school and below	25	1.82	
Marital status	Married	1059	77.30	–
	Unmarried	270	19.71	
	Divorced/widowed	41	2.99	
Professional title	Senior title	44	3.21	–
	Middle title	207	15.11	
	Junior title	724	52.85	
	No title	395	28.83	
Monthly income (RMB)	> 5000	55	4.01	–
	4001~5000	274	20.00	
	3001~4000	519	37.88	
	2001~3000	413	30.15	
	< 2000	109	7.96	
Hire form	Personnel agent staff	205	14.96	–
	permanent staff	221	16.13	
	Contract staff	185	13.50	
	Temporary staff	759	55.40	
Working time (hours a week) ^**b**^	< 30	18	1.31	42.92 (8.48)
	31–40	973	71.02	
	41–50	235	17.15	
	> 50	144	10.51	
Working years ^**b**^	1–5	404	29.49	14.65 (10.87)
	6–10	230	16.79	
	11–15	134	9.78	
	15–20	169	12.34	
	21–25	149	10.88	
	> 25	287	20.95	
Frequency of night shift (times a week)	0	868	63.36	–
	1–3	450	32.85	
	> 3	52	3.80	

Note: ^a^ SD, standard deviation, this indicator was calculated only for quantitative data. ^b^ represented quantitative data

### Job satisfaction scale

Job satisfaction was measured with 18 items selected from the Minnesota Satisfaction Questionnaire (MSQ) [31] and the Job Satisfaction Survey (JSS) [32]. The content of job satisfaction included satisfaction with environment, remuneration, management, the work itself [22, 33]. Sample item includes “The comfort level of the working environment (office environment, greening, lighting) will satisfy you.” (KMO measure =0.957, *p* < 0.01, Cronbach’s α = 0.970).

### Burnout scale

We used 5 items from the Maslach Burnout Inventory (MBI) [34] to measure individual burnout, and aggregated it to measure a positive effect on the burnout. Participants respond to the following items: “I feel that my daily [35] work is meaningless”, “I can’t find a sense of accomplishment at work”, “I feel exhausted when I get off work every day”, “this job has made me indifferent” and “this job makes me feel restless”. (KMO measure =0.857, *p* < 0.01, Cronbach’s α = 0.925).

### Turnover intention scale

The turnover intention questionnaire was designed with reference to turnover intention scale explored by Griffeth [36]. We measured turnover intention using 5 items. Participants respond to the following items: “I had the idea of leaving this organization”, “within a year, I will go to find a new job”, “If there is an opportunity, I will definitely accept a better job”, “I think the employment situation in this organization is very good” and “Currently, I agree to find a good job in the market”. (KMO measure =0.800, *p* < 0.01, Cronbach’s α = 0.721).

### Statistical analysis

Data entry and conversion was completed with EpiData 3.0. Double machine imputing method was used to enter the collected data into the computer. Descriptive analyses were conducted to describe social demographic factors. The structural equation model (SEM) was performed to adjust model fitting, the mediation effect test was carried out by using the bootstrap method. Sobel-Z test was used to verify the significance of mediation effect. Using SPSS 20.0 (IBM Corp, Armonk, NY, USA) and AMOS 24.0 (IBM Corp, Armonk, NY, USA) to analyze data, and *p* < 0.05 was determined to significant in statistics.

## Results

### Descriptive statistics

Table 1 showed the sociodemographic characteristics of the respondents. The mean age was 36.98 ± 9.84 years (minimum: 18 years, maximum: 73 years). 426 (31.09%) medical staffs were male and 944 (68.91%) were female. 42.77% of participants were physicians and 41.02% were nurses. The largest number of participants in the 40–49 age group, accounting for 34.45%, while the group over the age of 49 accounts for 11.31%. Most participants (77.30%) were married, 724 (52.58%) had a junior title and 63 % had no night shift in their work.

### Structural equation model constructing and fitting

The Hypothetical model was established, as shown in Fig. 1. We have constructed four paths: (1) Path a: Path from independent variable to potential mediator variable, the path coefficient of path a represents the indirect effect of burnout to job satisfaction (Job satisfaction ← Burnout). (2) Path b: The path from potential mediator variable to dependent variable, the path coefficient of path b represents the indirect effect of job satisfaction to turnover intention (Turnover intention ← Job satisfaction). (3) Path c: the path from independent variable to dependent variable, the path coefficient of path c represents the total effect of burnout to turnover intention (Turnover intention ← Burnout). (4) Path c’: Under the influence of potential mediator variables, the path from the independent variable to the dependent variable, the path coefficient of path c’ represents the direct effect of burnout to turnover intention (Turnover intention’ ← Burnout’).

**Fig. 2:**
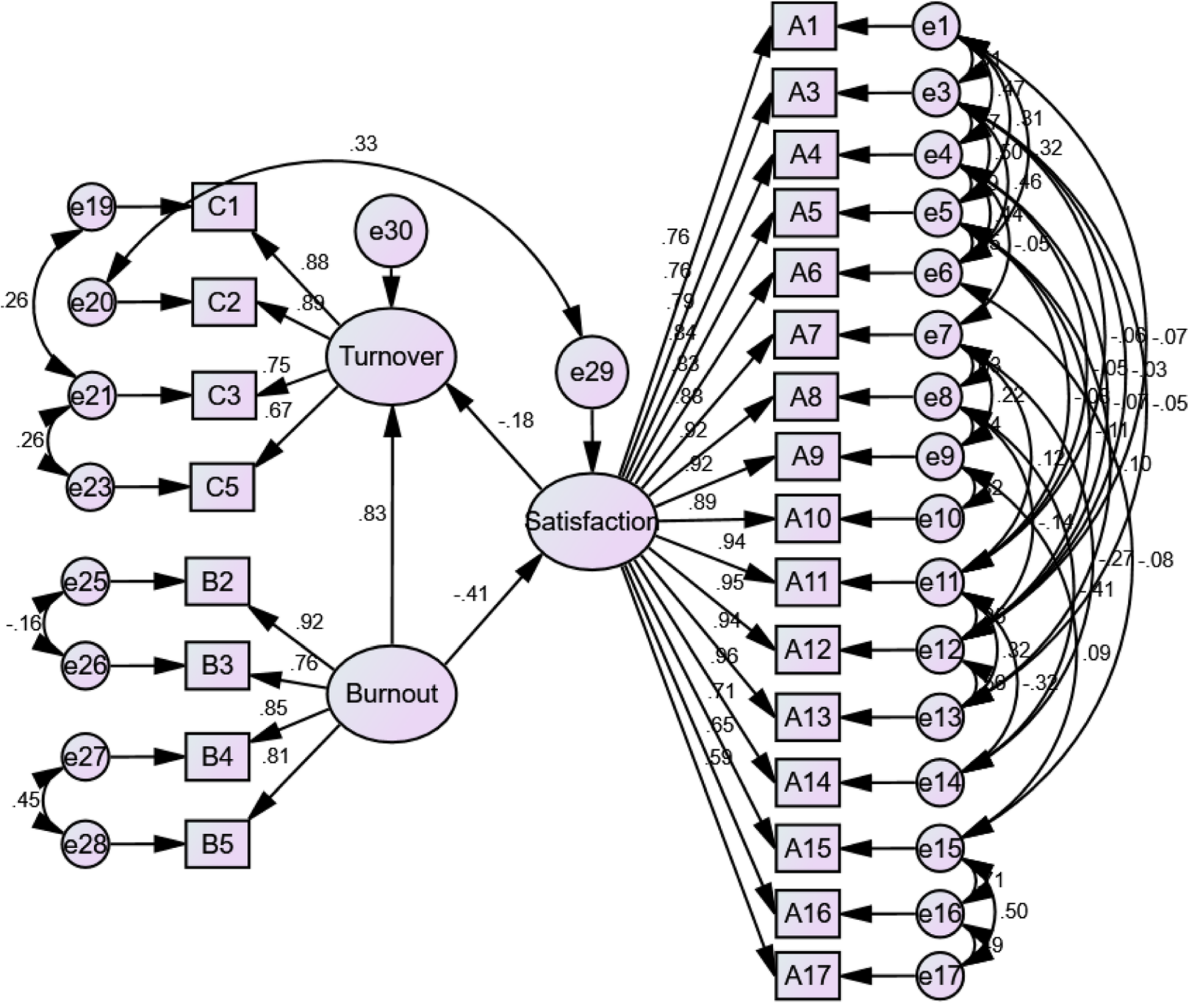
The revised structural equation modeling of the relationship between job satisfaction, burnout and turnover intention

As shown in Table 2. From the results of the hypothetical model operation, we found that all fitting indices did not meet the fitting criteria, indicated that the hypothetical model was not ideal, so we revised the model. The model path was modified according to the amendment advice given by AMOS. We removed some items (A18, B1 and C4) and added lots of bidirectional arrows to make the model fitting better. The final fitting indices results were also shown in Table 2, and the revised standardized path coefficient map was displayed on Fig. 2. After the adjustments, validity and reliability of the three scales remained acceptable. KMO measure, *p* value for batrtlett’s spherical test and Cronbach’s α for job satisfaction is 0.957, < 0.01 and 0.976, respectively; For turnover intention is 0.857, < 0.01 and 0.910, respectively; For burnout is 0.798, < 0.01 and 0.879, respectively.

**Table 2:** Comparison of different fitting indices on hypothetical model and adjusted model

Fit index	χ^2 a^	χ^2^/df ^b^	GFI ^c^	AGF ^d^	CFI ^e^	NFI ^f^	IFI ^g^	TLI ^h^	RMSEA ^i^	AIC ^j^
Optimum model	–	2–5	> 0.90	> 0.90	> 0.90	> 0.90	> 0.90	> 0.90	< 0.05	–
Hypothetical model	9695.143	27.940	0.600	0.533	0.801	0.796	0.802	0.784	0.140	9813.143
Results	–	unfit	unfit	unfit	unfit	unfit	unfit	unfit	unfit	–
Adjusted model	1157.059	5.590	0.932	0.901	0.977	0.973	0.977	0.970	0.058	1343.059
Results	–	acceptable	fit	fit	fit	fit	fit	fit	acceptable	–

Note: ^a^ χ2, Chi-square. ^b^ χ2/df, Chi-square divided by degree of freedom. ^c^ *GFI* Goodness of fit index, ^d^ *AGFI* Adjusted goodness of fit index, ^e^ *CFI* Comparative fit index, ^f^ *NFI* Normed fit index, ^g^ *IFI* Incremental fit index. ^h^ *TLI* Tucker-Lewis index. ^I^ *RMSEA* Root mean square error for approximation. ^j^ *AIC* Akaike information criterion

As shown in Table 3, we estimated the significance of total effect, direct effect and indirect effect by bias-corrected approach. The results showed that the total effect was significant of independent variable (burnout) to dependent variable (turnover intention) (*p* < 0.01), that was, the total effect of path c was statistically significant. The direct effects of path a (Job satisfaction Burnout) (*p* < 0.01), path b (Turnover intention ← Job satisfaction) (*p* < 0.01) and path c’ (Turnover intention’ ← Burnout’) (*p* < 0.01) were also significant. The results of indirect effect test again proved that path c’ (Turnover intention’ ← Burnout’) was statistically significant. We concluded that this mediating effect was a partial mediating effect.

**Table 3:** The *p*-value of significance test of total effect, direct effect and indirect effect by bias-corrected approach

		Burnout	Job satisfaction	Turnover intention
Total effect	Job satisfaction	0.001	–	
	Turnover intention	0.001	0.001	–
Direct effect	Job satisfaction	0.001	–	–
	Turnover intention	0.001	0.001	–
Indirect effect	Job satisfaction	–	–	–
	Turnover intention	0.001	–	–

As shown in Table 4, standardized estimates and its standard errors were calculated by using the bootstrap method. Standardized path coefficient of path a (Job Satisfaction ← Burnout) is − 0.410 and its standard error (S_a_) is 0.038. Standardized path coefficient of path b (Turnover intention ← Job satisfaction) is − 0.180 and its standard error (S_b_) is 0.028. Standardized path coefficient of path c’ (Turnover intention’ ← Burnout’) is 0.824 and its standard error (S_c′_) is 0.029. Looking up the related tables, the standardized path coefficient of path c is 0.899 and its standard error (S_c_) is 0.020. Soble-*Z* test is carried out according to the formula $\overline{z}=\frac{\mathrm{a}b}{\sqrt{{s_a}^2{b}^2+{s_b}^2{a}^2}}$. Finally, $\overline{\mathrm{z}}$ = 4.506. According to MacKinnon’s critical value table, the result is *p* < 0.05, indicating that the mediation effect is significant.

**Table 4:** Standardized path coefficient and standard error of three main paths by bootstrap

Path			SE	SE-SE	Mean	Bias	SE-Bias
Job Satisfaction	←	Burnout	0.038	0.001	−0.410	0.000	0.001
Turnover	←	Job Satisfaction	0.028	0.000	−0.180	−0.002	0.001
Turnover’	←	Burnout’	0.029	0.000	0.824	−0.002	0.001

Note: *SE* standard error. *SE-SE* standard error caused by using bootstrap to estimate standard errors. *SE-Bias* the standard error of bias

### Interpretation of the revised model

Model fit is acceptable if *χ*^2^/df ≤ 4.0 [37], GFI > 0.90, AGFI > 0.90, CFI > 0.90, NFI > 0.90, IFI > 0.90 [38], TLI > 0.90 and RMSEA < 0.05. As shown in Table 2, all fit indexes are up to standard, except for *χ*^2^/df and RMSEA. Our sample size is larger than 1000, the value of *χ*^2^/df is acceptable. In another study, the author points out that 0.05 < RMSEA < 0.08 is also acceptable [39]. Overall, the model fits well and the model is established.

As shown in Fig. 2, the standardized path coefficient of burnout to job satisfaction is − 0.41, indicates that burnout is negatively correlated with job satisfaction (*p* < 0.01). It shows that when the other conditions are unchanged, the turnover intention decreases by 0.41 units for each additional unit of burnout. The standardized path coefficient of job satisfaction to turnover intention is − 0.18, demonstrates that job satisfaction is also negatively correlated with turnover intention (*p* < 0.01). Under the same other conditions, the turnover intention decreases by 0.18 units for each additional unit of job satisfaction. The standardized path coefficient of burnout to turnover intention is 0.83, reveals that burnout is positively correlated with turnover intention (*p* < 0.01). That is, under the influence of job satisfaction, the turnover intention increases by 0.83 units for each additional unit of burnout.

The mediation effect is statistically significant (*p* < 0.01), and the impact of burnout on turnover intention through the intermediary effect of job satisfaction is 0.074 (a*b = (− 0.180)*-(0.410)). It manifests that when other conditions remain unchanged, the turnover intention will be indirectly increased by 0.074 units for each unit of burnout.

## Discussion

Our results demonstrated that for medical workers in primary care institutions, a mediator variable was existed in burnout and turnover intention: job satisfaction. Job satisfaction was usually regarded as a dependent variable [40–42] or an independent variable [43, 44] in most of the current studies. And work to family conflict [42], work engagement [40], burnout and workload [45] were viewed as mediator variables. However, an American study suggested that burnout was the biggest predictor of job satisfaction [13]. There were few researches focus on job satisfaction as a mediator variable, which provides new ideas for future research. That’s why we try to study how job satisfaction as a mediating variable affects the correlation between burnout and turnover intention.

The results of the study provided further support for the importance of job satisfaction in engaging the workforce and retaining staff to settle the demands and challenges facing health care setting in primary care institutions. Turnover intention was negatively related to job satisfaction and positively related to burnout. Negative correlation was found between job satisfaction and burnout. Some studies conducted in China have also obtained similar results [14, 15, 22–24, 26, 27, 46], but the correlation coefficient of their results is greater than ours. In our study, the correlation coefficient of path b (Turnover intention ← Job satisfaction) is relatively small, only − 0.18. We speculated that traditional studies which usually used methods such as multivariate linear regression, logistic regression, and ANOVA may neglect the measurement error, so its negative correlation is stronger. However, the error is taken into account in the SEM.

Our finding showed that the mediating effect was a partial mediating effect. First of all, we need to realize what the difference between partial mediation and complete mediation is. In the process of partial mediation, any variable in the causal chain, when it controls the variable before it (including the independent variable), it will affect its subsequent variables significantly. And in the process of complete mediation, after controlling the mediation variables, the influence of the independent variables on the dependent variables is not significant. Job burnout is closely related to turnover intention, and job burnout affects turnover intention directly [17–22], thus causing a partial mediating effect.

Although the mediation effect test confirms that the existence of job satisfaction as a mediating variable of job burnout affects turnover intention, the mediation effect has a low impact of 7.4%. That is to say, the mediation effect accounts for 7.4% of the variation of dependent variables. Despite the low mediation effect, we still believe that the research is valuable and meaningful. We speculated that the low mediation effect was caused by the following reasons. Firstly, it is related to the choice of independent variables closely. Job burnout includes three dimensions: emotional exhaustion, depersonalization and reduced personal accomplishment [6]. For example, family conflict and doctor-patient relationship are more intuitive than job burnout as important factors that directly affect emotions. If we take them as independent variables directly, the respondent would understand the meaning of the question more clearly, therefore, the path coefficients of path a (mediator variable ← independent variables) would be larger than those of the study (job satisfaction ← burnout), which will lead to a higher mediation effect. Secondly, turnover intention, burnout and job satisfaction are difficult to measure directly. The items and measuring methods may be somewhat various in different studies [47]. Therefore, the difference of instrument selection is also one of the important reasons for the inconsistency of results. Thirdly, the influence of job satisfaction on turnover intention is limited. There are many factors affecting turnover intention. Turnover intention is influenced not only by job satisfaction, but also by social demographic factors such as education [43], years in work [43], family’s relationship [43], monthly income, social support [48], mentoring [49], etc. and other unobserved factors. Therefore, these demographic factors and unobserved factors should be taken into account in the future study.

Our study also has some limitations. Firstly, Because of the diversity of the participants (including doctors, nurses, technicians.), we don’t use the international scale completely. According to the research purpose, the appropriate items have been chosen from these international scales, and the reliability and validity of the questionnaire are still guaranteed. Secondly, sample representativeness needs to be improved. This research can not be generalized to all china as our study place limited. In the future, we will continue to cooperate with other local governments in central China and to conduct similar surveys to solve the problem.

## Conclusions

Our study provides a clear understanding of how job satisfaction can mediate the relationship between burnout and turnover intention. In the process of burnout affecting turnover intention, job satisfaction can be regarded as a mediating variable to influence its effect, and the mediating effect was a partial mediating effect. And the mediation effect has a low impact of 7.4%. Turnover intention was negatively related to job satisfaction and positively related to burnout, job satisfaction was negative related to burnout. We can make full use of this relationship to adjust the impact of job burnout on turnover intention by improving job satisfaction. Some operable and useful measures were taken to reduce employee turnover rate and alleviating the current situation of shortage of health personnel in China, such as improving treatment and giving more promotion opportunities for workers to improve job satisfaction, conducting career planning courses and paying attention to employee psychological health to reduce job burnout.

## Supplementary information

**Additional file 1: Figure S1.** A flow diagram of participants.

**Additional file 2: Table S1.** Contents of three scales in the questionnaire.

## Data Availability

The data that support the findings of this study are available from the CDC of Huangpi district but restrictions apply to the availability of these data, which were used under license for the current study, and so are not publicly available. Data are however available from the authors upon reasonable request and with permission of the CDC of Huangpi district.

## Abbreviations

AGFI: Adjusted goodness of fit index
AIC: Akaike information criterion
ANOVA: Analysis of Variance
CFI: Comparative fit index
GFI: Goodness of fit index
IFI: Incremental fit index
JSS: Job Satisfaction Survey
MBI: Maslach Burnout Inventory
MSQ: Minnesota Satisfaction Questionnaire
NFI: Normed fit index
RMSEA: Root mean square error for approximation
S_a_: The standard error of standardized path coefficient of path a
S_b_: The standard error of standardized path coefficient of path b
S_c_: The standard error of standardized path coefficient of path c
S_c′_: The standard error of standardized path coefficient of path c’
SD: Standard Deviation
SE: Standard Error
SE-Bias: The standard error of bias
SEM: Structural Equation Model
SE-SE: The standard error caused by using bootstrap to estimate standard errors
TLI: Tucker-Lewis index
*χ*^2^: Chi-square
*χ*^2^/df: Chi-square divided by degree of freedom
