# Supplementary material for: Moderating role of job satisfaction on turnover intention and burnout among workers in primary care institutions: a cross-sectional study
Source: BMC Public Health. 2019 Nov 14;19:1526. doi: 10.1186/s12889-019-7894-7 (PMC6857324; doi:10.1186/s12889-019-7894-7)
Supplement: Supplementary file 1 — Additional file 1: Figure S1. A flow diagram of participants. [file 12889_2019_7894_MOESM1_ESM.docx]

Participants from all public medical institutions in rural Huangpi District (included 18 township health centers and 2 community health centers)

18 township health centers (n= 1259)

2 community health centers (n=123)

Participants not meet inclusion criteria were excluded (n=20)

18 township health centers (n= 1254)

2 community health centers (n=122)

18 township health centers (n= 1278)

2 community health centers (n=124)

Participants who were not in the post during the investigation were excluded (n=6)

18 township health centers (n= 1250)

2 community health centers (n=120)

Questionnaires with uncompleted answers (n=4) and suspected unreal answers were excluded (n=2)
