# Supplementary material for: Moderating role of job satisfaction on turnover intention and burnout among workers in primary care institutions: a cross-sectional study
Source: BMC Public Health. 2019 Nov 14;19:1526. doi: 10.1186/s12889-019-7894-7 (PMC6857324; doi:10.1186/s12889-019-7894-7)
Supplement: Supplementary file 2 — Additional file 2: Table S1. Contents of three scales in the questionnaire. [file 12889_2019_7894_MOESM2_ESM.docx]

*Table S1 Contents of three scales in the questionnaire*

| Scale | Content of each item |
| --- | --- |
| Job satisfaction | A1. The comfort of the working environment (office environment, greening, lighting). |
|  | A2. The organization can provide sufficient technical equipment (professional information inquiry resources, instruments) for work use. |
|  | A3. Interpersonal relationship within the unit (peer relationship and subordinate relationship). |
|  | A4. Harmonious cooperation between different departments within the unit. |
|  | A5. The work style of the unit. |
|  | A6. The leadership of the work unit. |
|  | A7. Satisfaction of current income. |
|  | A8. Satisfaction of the welfare of the work unit. |
|  | A9. Satisfaction of work development prospects |
|  | A10. Satisfaction with the training opportunities (frequency, form and content). |
|  | A11. The unit's income distribution system. |
|  | A12. The unit's performance appraisal mechanism. |
|  | A13. The performance of the unit's performance reward system. |
|  | A14. The management system and business process of the unit. |
|  | A15. I am very interested in my current job. |
|  | A16. My character and ability suit my current position. |
|  | A17. My work is very challenging. |
|  | A18. I feel that my daily work tasks are heavy. |
| Burnout | B1. I feel that my daily work is meaningless. |
|  | B2. I can't find a sense of accomplishment at work. |
|  | B3. I feel exhausted when I get off work every day. |
|  | B4. This job has made me indifferent. |
|  | B5. This job makes me feel restless. |
| Turnover | C1. I had the idea of leaving this organization. |
|  | C2. within a year, I will go to find a new job. |
|  | C3. If there is an opportunity, I will definitely accept a better job. |
|  | C4. I think the employment situation in this organization is very good. |
|  | C5. Currently, I agree to find a good job in the market. |
